# HPT: A High Spatial Resolution Multispectral Sensor for Microsatellite Remote Sensing

**DOI:** 10.3390/s18020619

**Published:** 2018-02-18

**Authors:** Junichi Kurihara, Yukihiro Takahashi, Yuji Sakamoto, Toshinori Kuwahara, Kazuya Yoshida

**Affiliations:** 1 Faculty of Science, Hokkaido University, Kita 10 Nishi 8, Kita-ku, Sapporo 060-0810, Japan; yukihiro@sci.hokudai.ac.jp; 2 Department of Aerospace Engineering, Tohoku University, Aramaki Aza Aoba 6-6-11, Sendai 980-8579, Japan; sakamoto@astro.mech.tohoku.ac.jp (Y.S.); kuwahara@astro.mech.tohoku.ac.jp (T.K.); yoshida@astro.mech.tohoku.ac.jp (K.Y.)

**Keywords:** multispectral sensor, tunable filter, microsatellite, Earth observation, image analysis

## Abstract

Although nano/microsatellites have great potential as remote sensing platforms, the spatial and spectral resolutions of an optical payload instrument are limited. In this study, a high spatial resolution multispectral sensor, the High-Precision Telescope (HPT), was developed for the RISING-2 microsatellite. The HPT has four image sensors: three in the visible region of the spectrum used for the composition of true color images, and a fourth in the near-infrared region, which employs liquid crystal tunable filter (LCTF) technology for wavelength scanning. Band-to-band image registration methods have also been developed for the HPT and implemented in the image processing procedure. The processed images were compared with other satellite images, and proven to be useful in various remote sensing applications. Thus, LCTF technology can be considered an innovative tool that is suitable for future multi/hyperspectral remote sensing by nano/microsatellites.

## 1. Introduction

Artificial satellites can be categorized according to their masses, e.g., picosatellites (<1 kg), nanosatellites (1–10 kg), microsatellites (10–100 kg), minisatellites (100–1000 kg), and large satellites (>1000 kg). With the rapid development of electronic and communication technology, the adage “smaller, faster, and cheaper” summarizes the trend of satellite development activities, which has resulted in a significant increase in the number of nano/microsatellites launched over recent years, especially for Earth observation and remote sensing purposes [1]. Nano/microsatellites have great potential as remote sensing platforms because of the cost-effective implementation of satellite constellations and formations, which increase their overall temporal resolution and ground coverage [2]. However, nano/microsatellites have many limitations related to their small size, which lead to restrictions on their payload instruments in terms of design, power consumption, and data rate. For optical remote sensing, the spatial and spectral resolutions of an optical payload instrument are limited primarily by its aperture size, which is constrained by the dimensions of the satellite. For any optical system, irrespective of whether it is a spaceborne or ground-based instrument, its spatial resolution is limited theoretically by the diffraction of light, and its spectral resolution is limited practically by the signal-to-noise ratio (SNR). Both the diffraction and the SNR can be upgraded with larger apertures; thus, nano/microsatellites have fundamental disadvantages compared with larger satellites in terms of optical remote sensing [3].

**Figure 1:**
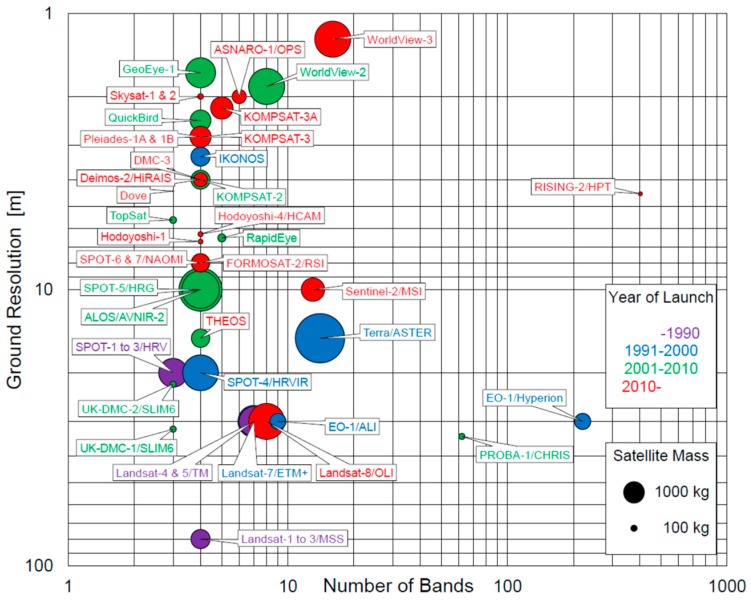
Diagram of the high and moderate spatial resolution multispectral Earth observation satellites/sensors launched in the past.

Figure 1 shows a diagram of the distribution of high (<10 m) and moderate (10–100 m) spatial resolution multispectral Earth observation satellites/sensors launched in the previous few decades. The mass of a satellite and the decade of its launch are indicated by the area and the color of the circle, respectively. As can be seen, satellite sensors with three or four spectral bands show a trend toward higher ground resolutions and smaller satellite sizes. Another evident trend is the increase in the number of spectral bands for a series of large satellites, such as the Landsat and WorldView flagship series. Aside from the multispectral Earth observation satellites, only two satellites, Earth Observing-1 (EO-1) and Project for On-Board Autonomy-1 (PROBA-1), are experimentally equipped with high spatial resolution hyperspectral sensors, although additional operational hyperspectral missions are planned for launch before 2020 [4].

Hyperspectral sensors, which are also known as imaging spectrometers, are capable of obtaining images in hundreds of spectral bands, and they can provide abundant information in relation to a wide variety of Earth observation and remote sensing applications. Although the challenges in adapting high spatial resolution hyperspectral sensors to small satellites have been studied theoretically in terms of the resolution, quality, and volume of hyperspectral data, the practical feasibility appears low for satellites <100 kg, even when applying state-of-the-art technology [5,6]. The hyperspectral sensor considered in the above studies was a push-broom/line-scanning spectrometer, which had been conventionally mounted on large satellites or on manned aircraft. This type of hyperspectral sensor has a two-dimensional (2D) detector array, where one axis records spatial information and the other axis records spectral information. The spatial axis is arranged perpendicular to the satellite track; thus, by scanning the Earth’s surface along the path of movement of the satellite, a three-dimensional (3D) hyperspectral data cube is obtained. Consequently, both the resolution and the quality of the acquired data are constrained considerably by the ground track velocity of the satellite and the aperture size of the sensor. These constraints make it difficult to realize effective deployment of high spatial resolution hyperspectral sensors on nano/microsatellites. 

Another type of multi/hyperspectral sensor is a wavelength-scanning imager using a filter wheel or a tunable filter. This type of sensor also employs a 2D detector array, but it captures a spatial 2D image filtered at a certain spectral band that is switched by mechanically rotating the filter wheel or by electrically tuning the filtered wavelength. While a finite response time is required to switch from one spectral band to another for wavelength scanning, the resolution and the quality of the data do not depend directly on the satellite speed. A filter wheel mounted with 15 spectral filters has already been applied to spaceborne low spatial resolution (>100 m) multispectral sensors, such as the Polarization and Directionality of the Earth’s Reflectances instrument on the Polarization and Anisotropy of Reflectances for Atmospheric Sciences coupled with Observations from a light detection and ranging (LiDAR) (PARASOL) microsatellite [7]. The disadvantage of a filter wheel in relation to small satellites is that the diameter, weight, and power consumption of the filter wheel all increase with the number of spectral bands.

Recently, we have developed a high spatial resolution multispectral sensor using a tunable filter to overcome the disadvantage of a filter wheel, and installed it on the RISING-2 microsatellite. This sensor, which is equipped with a liquid crystal tunable filter (LCTF) for wavelength scanning, is called the High-Precision Telescope (HPT). To the best of our knowledge, the HPT is the first spaceborne sensor using LCTF technology. The advantages for small satellites in using the LCTF are related not only to the reductions in the size, weight, and power consumption of the sensor, but also to the increased flexibility of the spectral bands and enhanced data volumes. The central wavelength of the spectral bands is electrically tunable for every image acquisition; hence, the data volume could be reduced by choosing appropriate spectral bands for specific purposes. This flexibility allows a single multispectral sensor on a nano/microsatellite to be applied to various remote sensing fields, making it comparable with hyperspectral sensors. As shown in Figure 1, the HPT holds a unique position amongst the current multispectral Earth observation sensors.

This paper presents descriptions of both the design and development of the HPT deployed on the RISING-2 microsatellite, and the data analysis procedure adopted for the acquired images.

## 2. Sensor Characteristics

### 2.1. RISING-2 Microsatellite

The RISING-2 microsatellite is a 50-kg platform developed jointly by the Tohoku and Hokkaido universities in Japan [8]. It constitutes a substantial improvement over the SPRITE-SAT, which was launched in 2009 and subsequently called the RISING satellite after its launch [9]. Tohoku University developed a satellite bus system for the RISING-2 platform, including a main onboard computer called the Satellite Central Unit (SCU). Hokkaido University developed the mission payload instrumentation for Earth observation, including a controller unit called the Science Handling Unit (SHU; manufactured by AD Co., Ltd., Sagamihara, Japan).

The RISING-2 microsatellite was designed with the form of a 50-cm cube and a weight of about 43 kg, such that it could be adopted as a secondary small payload on an H-IIA launch vehicle. The satellite was launched successfully by the H-IIA from Tanegashima Space Center in Japan on 24 May 2014, along with the primary payload (the Advanced Land Observing Satellite-2 satellite) and three other secondary small payloads (nano/microsatellites, each <50 kg). The RISING-2 microsatellite was placed into a Sun-synchronous orbit at the altitude of 628 km with the local Sun time on the descending node of 12:00. Three years after its launch, the satellite remains active, although its operation has become less frequent.

The three-axis active attitude control system of the RISING-2 microsatellite provides off-nadir pointing capability in order for the optical mission payload instruments to maintain focus on a specific location on the ground [10]. This capability not only enhances the revisit frequency for the selected site, it also increases the number of spectral bands available for the site by wavelength scanning with the LCTF of the HPT. The location of the site to be observed by the HPT is stored preliminarily in the memory of the SCU, and the HPT observation is executed at a scheduled time by sending the stored commands from the SCU to the SHU.

The SHU controls both the power supply and the data acquisition by the mission payload instruments. Acquired images are stored temporarily in the 8 MB Static Random Access Memory (SRAM) of the SHU. As the data volume of an image taken by the HPT is about 650 kB, the HPT can capture up to 12 images sequentially. As described later, the HPT has the potential for a hyperspectral sensor that is capable of obtaining images in hundreds of spectral bands, but it is not realized because of the limitation of the number of images stored temporarily. The SRAM allows fast access, but it is a volatile memory unit, and it loses stored data when the power is off. After an observation sequence, the images are combined with ancillary data, and transferred to the 128-MB non-volatile flash memory of the SHU without image data compression. When the RISING-2 microsatellite is visible from the ground station located at Tohoku University, the data in the flash memory are downlinked via S-band telemetry at a data rate of 50 kbps [8], which means that it takes at least 1.7 min to downlink one image. Therefore, because of this limitation of the data rate, fewer than 10 images can be downlinked in one day.

### 2.2. High Precision Telescope (HPT)

**Figure 2:**
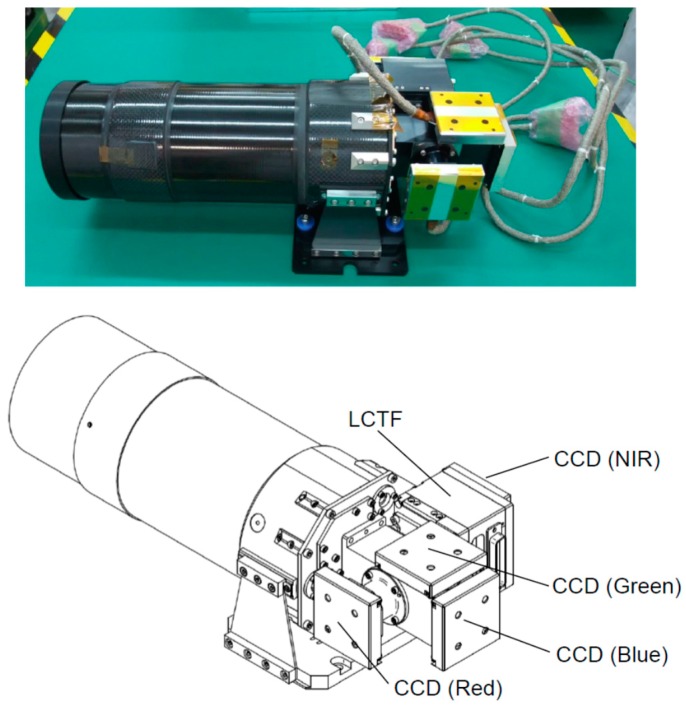
Appearance of the High-Precision Telescope (HPT).

**Figure 3:**
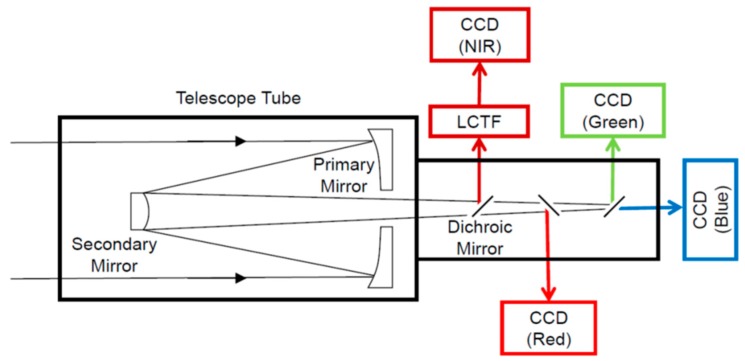
Schematic of the HPT.

The HPT installed on the RISING-2 microsatellite is a high spatial resolution multispectral sensor equipped with four charge-coupled device (CCD) image sensors for red, green, blue, and near-infrared (NIR) bands split by dichroic mirrors, as shown in Figure 2 and Figure 3. The LCTF is employed for wavelength scanning only in the NIR band, and it is placed in front of the NIR CCD module. The central wavelength of the NIR band is electrically selectable by controlling the LCTF for every image acquisition; thus, images of the spectral bands minimally required for specific purposes can be acquired sequentially. The specifications of the HPT are listed in Table 1.

The development of the HPT commenced in 2010, with the aim of achieving a high spatial resolution sensor suitable for deployment on a microsatellite for Earth observation. Earth observation has diverse requirements regarding spatial and spectral resolutions according to different fields of application, e.g., agriculture, environmental monitoring, hydrology, and oceanography (cf. Figure 2 in [2]). A multispectral sensor with 5-m spatial resolution can satisfy a wide range of these fields, except for intelligence services, traffic monitoring, and urban development purposes, which mostly require sub-meter resolution. Therefore, the goal of developing a 5-m-resolution multispectral sensor was envisioned for the HPT. In order to achieve a ground sample distance of 5 m with a pixel size of 7.4 μm from an altitude of 700 km, a focal length of 1 m is required for the optical system of the sensor. Considering the high SNR required for multispectral imaging, an aperture diameter of 100 mm is considered the largest possible for a 50-kg microsatellite. Accordingly, the diffraction-limited resolution on the focal plane of the optical system is 6.4 μm at the 500-nm wavelength. As the diffraction-limited resolution is close to the pixel size, a diffraction-limited optical system is required. This is why the name of the HPT sensor includes the word precision instead of the resolution.

The optical system of the HPT adopts a Cassegrain reflecting telescope that was designed and manufactured by Genesia Corp. (Mitaka, Japan). It is important in the design of an optical system for any spaceborne multispectral sensor to account for chromatic aberration and thermal stability. Compared with refracting telescopes using lenses, reflecting telescopes using mirrors have the advantage of no chromatic aberration, which is significant for wavelength scanning in a wide spectral range. Minimizing the chromatic aberration of a refracting telescope is possible, but balancing the chromatic aberration correction with thermal stabilization is usually difficult. The adoption of a reflecting telescope enables the development of the optical system to focus on thermal stability. In addition, the Cassegrain telescope comprises a parabolic primary mirror and a hyperbolic secondary mirror, which create the long focal length required for high spatial resolution imaging, while minimizing the length of the telescope tube by reflecting the light path. The telescope tube is made of carbon fiber-reinforced plastic, which has a very low coefficient of thermal expansion and high stiffness. The mirror is made from zero thermal expansion pore-free ceramics, which is a material developed by Nihon Ceratec Co., Ltd. (Sendai, Japan) for use in semiconductor manufacturing equipment. Although zero thermal expansion pore-free ceramics have an extremely low coefficient of thermal expansion at around room temperatures, active thermal control of the mirrors is impractical for the limited power supply of a microsatellite. Based on the results of thermal simulation analysis, both the tip of the telescope tube and the reverse side of the secondary mirror, which are exposed to the outside of the satellite, are covered by multilayer insulation to maintain the mirrors at room temperature passively. At room temperature, the Strehl ratio of the optical system measured with a laser interferometer is 0.92, which satisfies the criterion for a diffraction-limited system (>0.8).

A monochromatic CCD image sensor module is used for imaging in the four spectral bands. Generally, CCD image sensors consume more power, but are more sensitive than complementary metal oxide semiconductor (CMOS) image sensors. The most important characteristic of a CCD image sensor for satellite imaging is a global shutter that can capture the entire frame at the same instant, although many CMOS image sensors use a rolling shutter. The CCD module T065 manufactured by Watec Co., Ltd. (Tsuruoka, Japan) was developed originally for the optical mission payload instrumentation of the SPRITE-SAT. The unit cell size of this CCD module is 7.4 µm× 7.4 μm, and the effective image size is 659 × 494 pixels. The gain and exposure time of the CCD module are changeable from −6 to +36 dB and from 20 μs to 34 s, respectively, enabling the HPT to be applied to a wide range of spectral radiances on the Earth’s surface. An important function supported by this CCD module is the external trigger shutter that allows the synchronization of the three CCD modules for the blue, green, and red bands, in order to acquire a true color composite image.

**Figure 4:**
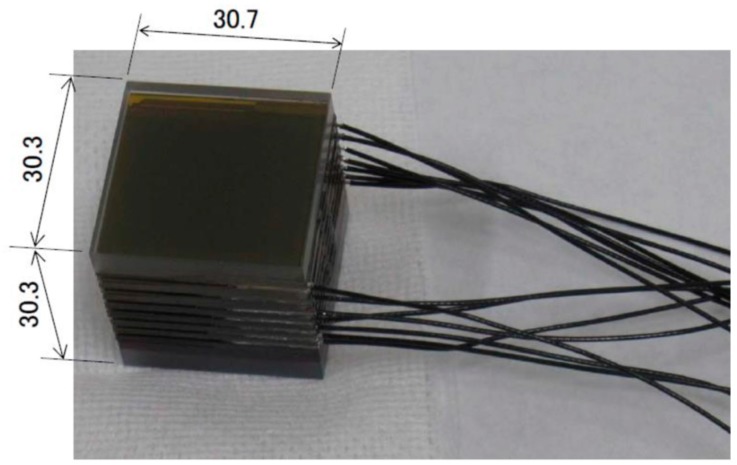
Photograph of the liquid crystal tunable filter (LCTF) (unit: mm).

In the HPT on the RISING-2 microsatellite, LCTF technology was applied for the first time to a spaceborne multispectral sensor through collaboration with the Research Institute for Advanced Liquid Crystal Technology at the Aomori Support Center for Industrial Promotion in Japan. The LCTF is a type of optical band pass filter that uses liquid crystal for the birefringent plates of the Lyot filter, and the LCTF technology has been used for many applications, e.g., agriculture, healthcare, archaeology, and art (see review by [11] and references therein). The LCTF is composed of several stacked layers of liquid crystal sandwiched between crossed polarizers, and the transmission wavelength of the LCTF is controlled by square-wave voltages that are applied to each layer. On the RISING-2 microsatellite, the LCTF and the voltage-controlling circuit are separated into the HPT and the SHU, respectively. The LCTF developed for the HPT (Figure 4) has dimensions of 30.7 mm × 30.3 mm × 30.3 mm, and it weighs 80 g. The power consumption of the voltage-controlling circuit is 0.2 W. These features enhance the compatibility of the LCTF technology with nano/microsatellites. The LCTF is used for wavelength scanning in the NIR band, and the central wavelength of the NIR band is electrically selectable at 1-nm intervals from 650 nm to 1050 nm. In terms of the number of spectral bands, the HPT has 401 bands in the NIR. The peak transmittance and the full width at half maximum increase with the central wavelength from 7% and 12 nm (at 650 nm) to 29% and 30 nm (at 1050 nm), respectively. To maintain the LCTF performance over a wide temperature range, a temperature sensor is attached to the LCTF, and the optimum voltages at the measured temperature are applied automatically using a look-up table stored in the voltage-controlling circuit. The response time for switching from one central wavelength to another depends on the temperature and on the combination of wavelengths. For example, the response time at 25 °C ranges from 39 ms (for a switch from 850 nm to 840 nm) to 259 ms (for a switch from 710 nm to 970 nm), and it is slower/faster at lower/higher temperatures, respectively. The exposure of the NIR CCD is designed to start after sufficient time has elapsed for the switching of the wavelengths.

## 3. Image Analysis Methods and Results

### 3.1. Color Images

A true color composite image is synthesized from red, green, and blue (RGB) band images captured synchronously by the HPT. The fields of view of the three bands deviate spatially from one another because of the optical alignment of their CCD image sensor modules. While the CCD modules are aligned precisely along the optical axis by the focus adjustment, they have an alignment precision of ~100 μm in the direction perpendicular to the optical axis, which corresponds to a deviation of a few tens of pixels. Therefore, band-to-band registration using a feature-based method is implemented in the image processing procedure. The scale-invariant feature transform method [12] is used for feature matching between the blue and green, and between the blue and red band images. From a single set of the two band images for a certain scene, their matched feature points are limited in number, and they are localized within the frame because of the difference in the spectral radiance of the different spectral bands. The matched feature points, which are usually found at the edges of black or white objects and clouds, are accumulated from many sets of different scenes, and a homography matrix is estimated from all of the matched feature points. The homography matrix describes a transformation that maps points in one plane to the corresponding points in another plane, and it is widely used for georeferencing. Based on each estimated homography matrix, the green and red band images are transformed into the coordinate system of the blue band image. Once a sufficiently precise transformation is accomplished, the same homography matrix can be applied to any other RGB band images.

**Figure 5:**
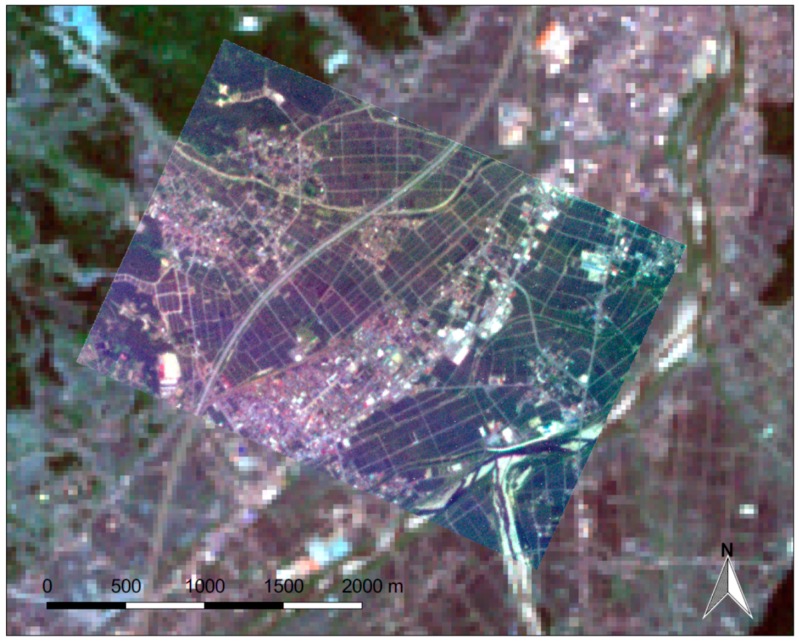
A true color composite image of Minami-Uonuma City, Niigata Prefecture, Japan, acquired by the RISING-2 HPT on 2 July 2014, overlaid on a true color composite image acquired by the Landsat 7 Enhanced Thematic Mapper Plus (ETM+) on 30 May 2014.

Figure 5 shows a true color image composed of the RGB bands selected from the “first light” scenes, which were acquired about one month after the launch of the RISING-2 microsatellite. For comparison, a true color image composed of Bands 1, 2, and 3 of the Enhanced Thematic Mapper Plus (ETM+) sensor on the Landsat 7 satellite with 30-m spatial resolution is also shown in Figure 5. The area in the scene is famous for its production of rice in Japan, and many rectangular paddy fields roughly 100 m × 50 m in size are visible in the image. Agricultural roads that are typically a few meters wide, which separate the paddy fields, are evident in the RISING-2 HPT image, but invisible in the Landsat 7 ETM+ image. This image demonstrates clear evidence of the 5-m resolution targeted by the HPT.

### 3.2. Multispectral Images

Multispectral images selectable from 650–1050 nm are acquired with the NIR band of the HPT. A combination of the selected wavelengths is configured in advance, and multispectral images are captured sequentially at a scheduled time and at a specified time interval. 

**Figure 6:**
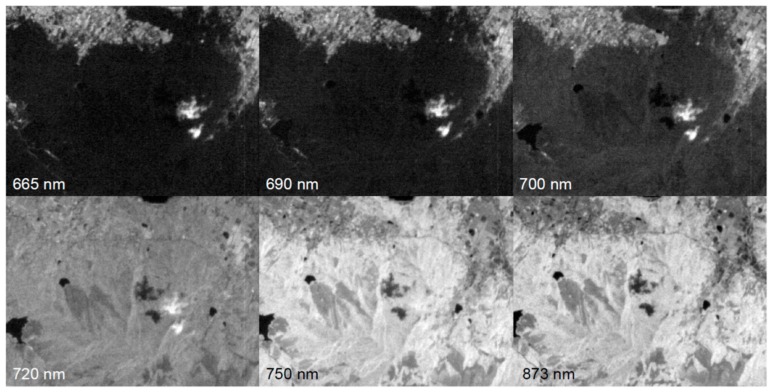
Multispectral images of Shimonoseki City, Yamaguchi Prefecture, Japan, acquired on 29 May 2015.

Figure 6 shows a series of multispectral images acquired at 665 nm, 690 nm, 700 nm, 720 nm, 750 nm, and 873 nm. The area in these scenes is dominated by forests, whose spectral reflectance is lower at 665 nm and 690 nm (the red region), and higher at 750 nm and 873 nm (the NIR region). Due to attitude fluctuations during the off-nadir pointing of the satellite, these scenes deviate from one another spatially. Hence, band-to-band registration using the feature-based method is also required for the processing of the multispectral images prior to the calculation of indices such as the normalized difference vegetation index (NDVI), which is derived from red and NIR images. The NDVI is calculated from the following expression:

(1) $$NDVI=\frac{\rho_{\mathrm{NIR}}-\rho_{\mathrm{RED}}}{\rho_{\mathrm{NIR}}+\rho_{\mathrm{RED}}}$$

where $\rho_{\mathrm{RED}}$ and $\rho_{\mathrm{NIR}}$ are the surface reflectance in the red and NIR regions, respectively. However, the result of feature matching between the red and NIR images is usually poor because of the differences in their spectral reflectance. As seen in Figure 6, the multispectral images at 720 nm, belonging to “red edge” between the red and NIR regions, have features common to both regions, and sufficient numbers of matched feature points. Therefore, the feature matching of multispectral images is iterated sequentially, with its neighboring spectral band from the red to the NIR through the red edge, before all of the images are transformed to the coordinate system of the NIR image. This spectrally sequential image registration method is also effective for the analysis of hyperspectral images obtained by a wavelength-scanning imager on an unmanned aerial vehicle [13].

**Figure 7:**
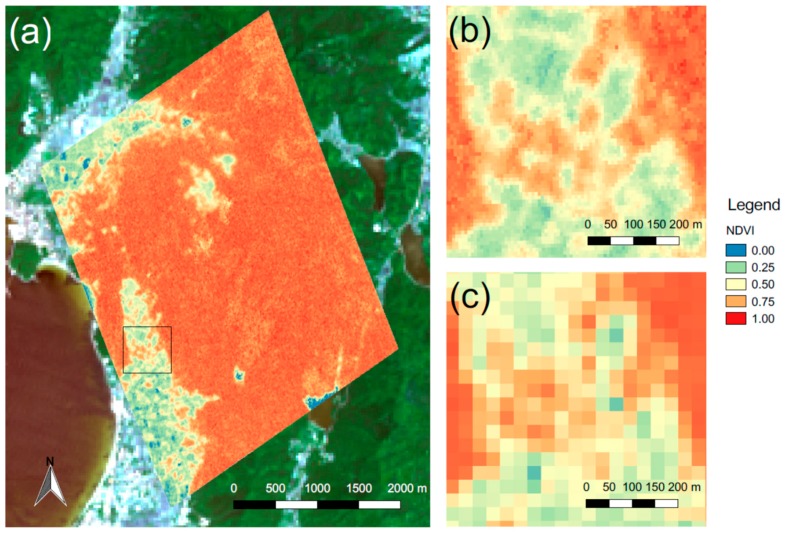
(**a**) Normalized difference vegetation index (NDVI) values derived from the multispectral images in Figure 6 overlaid on a true color composite image acquired by the Landsat 8 Operational Land Imager (OLI) on 21 May 2015, (**b**) enlarged view of the area framed by the black square in (**a**,**c**) NDVI values derived from the Landsat 8 OLI bands for the same area.

In order to calculate the NDVI, 10-bit digital numbers for each pixel after the image registration are converted to surface reflectance ρ using the following steps. (1) The digital number is converted into spectral radiance at the sensor’s aperture $L_{\mathrm{sat}}$ using the unit conversion coefficient, which is obtained by the preflight calibration test using an integrating sphere as the uniform light source. (2) An image-based atmospheric correction is applied to the path radiance $L_{p}$ calculation using the dark object subtraction (DOS) method [14,15]. (3) The surface reflectance is calculated as:

(2) $$\;\rho \;=\frac{\pi \;\left(L_{\mathrm{sat}}{\;-\;L}_{p}\right)\;d^{2}\;}{E\;\cos\theta }$$

where d is the Earth–Sun distance, E is the solar exoatmospheric irradiance, and θ is the solar zenith angle. Figure 7a,b shows the NDVI derived from the multispectral images in Figure 6. Images at 665 nm and 873 nm were used for the calculation of spectral reflectance in the red and NIR regions, respectively. A true color image comprising Bands 2, 3, and 4 of the Operational Land Imager (OLI) on the Landsat 8 satellite is also shown in Figure 7a for comparison. Figure 7c shows the NDVI derived from Bands 4 and 5 of the Landsat 8 OLI; this value was acquired eight days before the acquisition of the RISING-2 HPT images, which were corrected similarly using the DOS method. Although the spatial resolution of the RISING-2 HPT in this scene is 7.2 m and approximately four times higher than 30-m spatial resolution of the Landsat 8 OLI, the NDVI values in Figure 7b are in good agreement with those in Figure 7c. This image indicates that the multispectral image analysis was implemented successfully, and that the RISING-2 HPT was calibrated accurately during the preflight tests.

## 4. Future Research

Although the HPT project on the RISING-2 microsatellite was a pilot study, the results achieved were utilized for next-generation satellites. Multispectral sensors using LCTF technology have been developed further at Hokkaido University, and installed on subsequent microsatellites, e.g., the DIWATA-1, DIWATA-2, MicroDragon, and Rapid International Scientific Experiment Satellite (RISESAT). In these satellite sensors, a LCTF covering the visible spectral region is used in conjunction with that for the NIR. Over the past seven years, since the HPT project started, other related technologies have also been improved. The capacities of the volatile and non-volatile memories in the SHU have been increased up to 256 MB and 32 GB, respectively, and the downlink data rate with X-band telemetry has increased up to 10 Mbps. These technological developments have made multispectral remote sensing with LCTF technology more effective and operational. In fact, the DIWATA-1 microsatellite, which was launched on 23 March 2016, now routinely acquires multispectral images of several spectral bands over selected sites in the Philippines. The MicroDragon satellite will be launched in 2018, and will operate over Vietnam for ocean-color remote sensing, which requires at least eight spectral bands from the visible to NIR spectral regions [16]. The RISESAT [17], which is intended for launch together with the MicroDragon satellite, will have 5-m resolution (or higher) imaging in both the visible and the NIR regions. The image analysis methods presented in this paper can be utilized for images acquired by the above microsatellites that will be operated by members of the Asian microsatellite consortium, which was established in 2016 to share microsatellite technologies, observation data, and data application methods between its members.

## 5. Conclusions

The HPT on the RISING-2 microsatellite was developed to demonstrate the efficacy of high resolution multispectral remote sensing nano/microsatellite platforms. LCTF technology was applied for the first time to realize a compact multispectral spaceborne sensor. The advantages offered by the LCTF technology are reductions in the size, weight, and power consumption of the sensor, the flexibility of the spectral bands and data volume, and compatibility with off-nadir pointing. The RGB color and NIR multispectral images were acquired successfully by the HPT and processed by image analysis methods. LCTF technology, as demonstrated by its application in the HPT on the RISING-2 microsatellite, can be considered an innovative tool suitable for future multi/hyperspectral remote sensing by nano/microsatellites.

## Figures and Tables

**Table 1:** Specifications of the HPT.

Size	380 mm × 161 mm × 124 mm
Weight	3 kg
Focal length	1000 mm
Aperture diameter	100 mm
Ground sample distance	4.6 m (at nadir from 628 km altitude)
Field of view	0.28° × 0.21° (3.1 × 2.3 km at nadir from 628 km altitude)
Spectral bands	Blue: 400–510 nm Green: 520–600 nm Red: 610–650 nm NIR: 401 bands selectable at 1-nm intervals from 650 to 1050 nm
Image size	659 × 494 pixels
Data quantization	10 bit
